# Survival after initial lung metastasectomy for metastatic colorectal cancer in the modern chemotherapeutic era

**DOI:** 10.1186/s12893-017-0252-8

**Published:** 2017-05-10

**Authors:** Shintaro Yokoyama, Masahiro Mitsuoka, Tetsushi Kinugasa, Toshihiro Hashiguchi, Ryoichi Matsumoto, Daigo Murakami, Tatsuya Nishi, Koichi Yoshiyama, Masaki Kashihara, Shinzo Takamori, Yoshito Akagi

**Affiliations:** Department of Surgery, Kurume University School of Medicine, 67 Asahi-machi, Kurume, Fukuoka 830-0011 Japan

**Keywords:** Colorectal cancer, Lung metastasectomy, Prognostic factor, Recurrence, Survival

## Abstract

**Background:**

A clear survival benefit has been reported for lung metastasectomy for colorectal cancer, and several clinicopathological prognostic factors have been proposed in the past. However, clinical advances, such as chemotherapy and radiographic imaging, should have improved patient outcome and may have altered prognosticators. This study aimed to assess patient survival and determine prognostic factors for survival and recurrence in patients who underwent initial lung metastasectomy for colorectal cancer in the modern clinical era.

**Methods:**

Clinicopathological data and outcomes of 59 patients who underwent curative initial lung metastasectomy for colorectal cancer from 2004 to 2012 at a single institution in Japan were retrospectively investigated. Survival was estimated using the Kaplan - Meier method, and Cox proportional hazards regression models were used to estimate the prognostic impacts of each variable in univariate and multivariate analysis.

**Results:**

The 5-years overall and disease-free survival rates were 54.3 and 40.6%, respectively. A disease-free interval < 24 months after colorectal cancer resection (*P* = 0.004) and a serum carcinoembryonic antigen ≥ 5.0 ng/mL before initial lung metastasectomy (*P* = 0.015) were independent predictors for poor overall survival. Moreover, the disease-free interval after colorectal cancer resection < 24 months (*P* = 0.010) and a colorectal cancer with N2 stage disease (*P* = 0.018) were independently associated with poor disease-free survival. On the other hand, the number of lung metastasis was not identified as a poor prognostic factor for both overall and disease-free survival.

**Conclusions:**

Our findings demonstrated similar or slightly better overall survival, and substantially favorable disease-free survival as compared with past reports. Poor prognostic factors for overall survival appeared not to differ from those of past studies, although this modern series did not determine the number of lung metastasis as a poor prognostic factor, which should be investigated in future studies. Moreover, initial lung metastasectomy is not expected to be a curable treatment for patients with both a short disease-free survival after colorectal cancer resection and colorectal cancers with N2 stage disease.

## Background

Lung metastasectomy for colorectal cancer (CRC) has been shown to be an effective therapeutic strategy [1]. Currently, it is accepted worldwide as superior to non-surgical treatments for improving survival in patients with confined lung metastasis, with a concept of semi-local metastases [2]. The overall 5-years survival after lung metastasectomy is reported to be 22 – 68%, with a trend towards improved survival since 2000 [3–7]. Several previous studies have analyzed the post-lung metastasectomy prognostic factors, but no consensus has been reached, because of differences in the study findings. Therefore, although a prospective randomized trial is in progress, it is still not clear which patients will benefit most from lung metastasectomy [8].

The recent development of antitumor chemotherapeutic agents, such as irinotecan and oxaliplatin, has considerably prolonged survival in stage IV CRC patients [9]. Molecularly targeted drugs, which interrupt either epidermal growth factor receptor or vascular endothelial growth factor, have also yielded remarkable prognostic improvement [10, 11]. Currently, these antitumor agents are concomitantly administered as standard therapies for advanced CRC patients [10–13]. Moreover, these advances, along with surgical resection in selected patients, have allowed patients to attain a long disease-free survival, including patients with stage IV CRC. Furthermore, the development of high-resolution computed tomography (CT) for detecting extremely small metastases has allowed more accurate and complete resection, which may also have contributed to an improved prognosis. Therefore, clinicians should be aware of the significant effect these advances may have on the prognosis of patients who have undergone lung metastasectomy.

Against this background, we here assessed patient survival and determined prognostic factors for survival and recurrence in patients who underwent curative initial lung metastasectomy for CRC since 2004, at which time modern chemotherapeutic agents for CRC came to be used.

## Methods

### Patients

A total of 91 patients with lung metastases from CRC were considered candidates for lung metastasectomy in the Department of Surgery, Kurume University Hospital, between 2004 and 2012. Lung metastasectomy was performed in 66 of these patients, and the remaining 25 patients received chemotherapy or supportive care due to patient refusal, or because they had insufficient cardiopulmonary function for surgery. Two patients with incomplete resection and 5 patients with a history of lung metastasectomy for CRC metastasis were excluded; ultimately, 59 patients were included in the survival analysis.

Clinicopathological characteristics were obtained through review of the patients’ medical charts. All patients had undergone periodic clinical follow-up, at least every 6 months after surgery, including a serum carcinoembryonic antigen (CEA) level and a CT assessment. In cases of multiple lung metastases, the diameter of the largest tumor was used as the tumor size. The most extensive procedure was adopted for the analysis in cases in which combined operative procedures were performed for multiple lung metastases (e.g., if lobectomy and wedge resection had been performed, the case was enrolled as a lobectomy case). According to pathological features, each histological diagnosis was constructed based on the 2010 World Health Organization classification [14]. Microscopic negative surgical margins were confirmed in all of the resected lung tissues as well as in primary CRCs and liver metastases. Evaluation of venous invasion was performed by experienced pathologists using Elastica van Gieson staining as necessary.

The present study conforms to the tenets of the Declaration of Helsinki, and was approved by the Research Ethics Committee of Kurume University. Written informed consent was obtained from all of the patients.

### Surgical criteria

In our institution, lung metastasectomy for CRC metastases have been performed with curative intent. Surgical criteria included absence of other distant metastasis, excluding resectable hepatic metastasis. Patients with peritoneal disseminations or abdominal para-aortic lymph node metastases diagnosed using CT and 18^F^-fluorodeoxyglucose positron emission tomography (18^F^-FDG PET) were also excluded. In cases with mediastinal lymph nodes that were suspicious of metastasis on CT and 18^F^-FDG PET, endobronchial ultrasound-guided transbronchial needle aspiration biopsy was routinely performed in our institution; however, those cases were not included in the present study. All patients had adequately tolerable cardiopulmonary function, and all patients gave consent for lung metastasectomy.

### Statistical analysis

In survival analyses, the start point was defined as the day of lung metastasectomy. The end-point of the overall survival (OS) period was defined as the day of last follow-up or death caused by CRC, and that of the disease-free survival (DFS) period was the day when recurrence was confirmed at any site using CT, respectively. The definition of the disease-free interval (DFI) after CRC resection was established as follows. (1) In cases without a history of hepatic metastasis, DFI was defined as the duration between the day of primary CRC resection and the day when a lung metastasis was detected with CT. (2) In cases with a history of hepatic metastasis, the start-point was defined as the day when the resection of both primary CRC and hepatic metastasis had been completed, and the end-point was the day when a lung metastasis was confirmed using CT. All patients with both liver and lung metastatic lesions in this series, prior to lung metastasectomy, underwent liver metastasectomy or confirmed disappearance of the liver metastatic lesions after systemic chemotherapy, to ensure safe lung metastasectomy, particularly with regard to pulmonary function during general anesthesia.

The Kaplan - Meier methods were applied to evaluate survival distributions, and each curve was compared by the log-rank test. Cox proportional hazards regression models were adopted to estimate the prognostic values of each variable in univariate and multivariate analyses. A *P-*value < 0.05 was recognized as representing statistical significance. All statistical analyses were performed using JMP, version 11 software (SAS Institute, Cary, NC).

## Results

### Survival and prognostic factors affecting overall and disease-free survival after initial lung metastasectomy

**Fig. 1:**
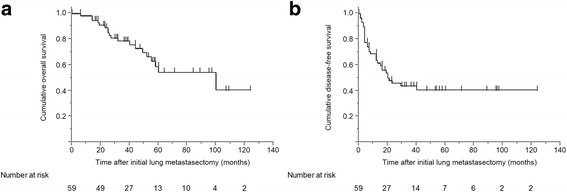
Kaplan - Meier survival curves for (**a**) overall survival and (**b**) disease-free survival in patients who underwent curative initial lung metastasectomy for colorectal cancer

The postoperative morbidity rate was 3.4%; this involved prolonged air leakage by alveolar fistula in 2 patients. No patients experienced postoperative hospital deaths. The median follow-up period was 38.0 months (range: 0 – 124). The cumulative 5-years OS rate was 54.3%, with a median survival time (MST) of 100.0 months (Fig. 1a). Univariate analysis indicated that male sex (*P* = 0.009), a serum CEA ≥ 5.0 ng/mL before lung metastasectomy (*P* = 0.023), and a DFI < 24 months after CRC resection (*P* = 0.006) predicted poor survival (Table 1).

**Table 1:** Univariate analysis of risk factors influencing overall and disease-free survival

Variable	*n* = 59 (%)	Overall survival			Disease-free survival		
		HR	[95% CI]	*P*	HR	[95% CI]	*P*
*General factors*							
Sex							
Female	26 (44)	1			1		
Male	33 (56)	3.780	[1.367–13.290]	0.009	2.076	[1.018–4.480]	0.045
Age (years old)							
≥ 70	20 (34)	1			1		
< 70	39 (66)	1.063	[0.417–3.042]	0.902	1.215	[0.595–2.625]	0.599
*Primary colorectal cancer related factors*							
Location							
Colon	34 (58)	1			1		
Rectum	25 (42)	2.524	[0.991–6.877]	0.052	1.725	[0.854–3.483]	0.127
Histological type/differentiation							
Well differentiated	21 (36)	1			1		
Moderately/poorly differentiated or mucinous	38 (64)	1.757	[0.634–6.188]	0.295	1.053	[0.520–2.273]	0.889
Preoperative serum CEA (ng/mL)							
< 5.0	23 (39)	1			1		
≥ 5.0	36 (61)	1.822	[0.660–6.400]	0.262	1.571	[0.751–3.586]	0.238
T stage							
T1/T2/T3	34 (58)	1			1		
T4	25 (42)	1.485	[0.598–3.740]	0.390	1.403	[0.690–2.823]	0.344
N stage							
N0/N1	50 (85)	1			1		
N2	9 (15)	2.839	[0.897–7.733]	0.073	3.496	[1.458–7.540]	0.007
Lymphatic invasion							
Not identified	19 (32)	1			1		
Identified	40 (68)	1.448	[0.520–5.117]	0.500	1.433	[0.670–3.410]	0.366
Vascular invasion							
Not identified	12 (20)	1			1		
Identified	47 (80)	3.555	[0.729–64.051]	0.135	3.412	[1.210–14.264]	0.018
pTNM stage							
Stage 1/2	29 (49)	1.351	[0.531–3.397]		1.022	[0.506–2.065]	
Stage 3/4	30 (51)	1		0.520	1		0.949
Adjuvant chemotherapy							
Not performed	28 (48)	1			1		
Performed	31 (53)	1.396	[0.558–3.769]	0.480	1.130	[0.609–2.483]	0.568
*Lung metastasis related factors*							
Number of tumors							
Solitary	48 (81)	1			1		
Multiple	11 (19)	1.496	[0.482–3.928]	0.456	1.908	[0.801–4.081]	0.136
Tumor size (mm)							
< 20	27 (46)	1			1		
≥ 20	32 (54)	1.985	[0.779–5.674]	0.154	1.591	[0.786–3.360]	0.199
Tumor distribution							
Unilateral	51 (86)	1			1		
Bilateral	8 (14)	2.376	[0.763–6.256]	0.126	2.528	[0.934–5.805]	0.066
Preoperative serum CEA (ng/mL)							
< 5.0	36 (61)	1			1		
≥ 5.0	23 (39)	3.030	[1.173–7.999]	0.023	2.230	[1.103–4.508]	0.026
Operative procedure							
Lobectomy	19 (32)	1			1		
Wedge resection/segmentectomy	40 (68)	1.911	[0.691–6.721]	0.225	1.049	[0.509–2.320]	0.899
DFI after colorectal cancer resection (months)							
≥ 24	20 (34)	1			1		
< 24	39 (66)	5.402	[1.545–34.105]	0.006	2.428	[1.105–6.096]	0.026
Adjuvant chemotherapy							
Not performed	33 (56)	1			1		
Performed	26 (44)	1.916	[0.772–4.974]	0.160	1.461	[0.725–2.945]	0.285
*Liver metastasis related factor*							
History of hepatic metastasis							
Absent	31 (53)	1			1		
Present	28 (48)	1.616	[0.647–4.356]	0.307	1.249	[0.620–2.518]	0.530

*CEA* carcinoembryonic antigen, *CI* confidence interval, *DFI* disease-free interval, *HR,* hazard ratio

With regard to DFS, the survival rate after 5 years was 40.6% with an MST of 20.0 months (Fig. 1b). Univariate analysis revealed male sex (*P* = 0.045), a primary CRC with N2 disease (*P* = 0.007), identification of vascular invasion by the primary CRC (*P* = 0.018), a serum CEA ≥ 5.0 ng/mL before lung metastasectomy (*P* = 0.026), and a DFI < 24 months after CRC resection (*P* = 0.026) as significant risk factors for recurrence (Table 1).

### Multivariate analysis for overall and disease-free survival

A multivariate model was used to identify prognostic factors for survival and recurrence (Table 2). A serum CEA ≥ 5.0 ng/mL before lung metastasectomy and a DFI < 24 months after CRC resection were shown to be independent poor prognostic factors for OS (*P* = 0.015 and 0.004, respectively). On the other hand, a primary CRC with N2 disease and a DFI < 24 months after CRC resection were identified as independent risk factors for recurrence (*P* = 0.018 and 0.010, respectively).

**Table 2:** Multivariate analysis of risk factors associated with overall and disease-free survival

Variable (risk)	HR	[95% CI]	*P*
Overall survival			
*General factor*			
Sex (male)	2.262	[0.746–8.336]	0.153
*Primary colorectal cancer related factors*			
Location (rectum)	2.042	[0.698–6.344]	0.193
N stage (N2)	1.422	[0.402–4.455]	0.566
*Lung metastasis related factors*			
Preoperative serum CEA (≥5.0 ng/mL)	3.793	[1.292–11.955]	0.015
DFI after colorectal cancer resection (<24 months)	6.424	[1.708–42.585]	0.004
Disease-free survival			
*General factor*			
Sex (male)	1.929	[0.893–4.352]	0.095
*Primary colorectal cancer related factors*			
N stage (N2)	3.106	[1.232–7.231]	0.018
Vascular invasion (identified)	2.395	[0.769–10.536]	0.141
*Lung metastasis related factors*			
Tumor distribution (bilateral)	2.227	[0.720–6.147]	0.156
Preoperative serum CEA (≥5.0 ng/mL)	1.977	[0.869–4.462]	0.103
DFI after colorectal cancer resection (<24 months)	2.838	[1.271–7.224]	0.010

*CEA* carcinoembryonic antigen, *CI* confidence interval, *DFI* disease-free interval, *HR* hazard ratio

### Survival analysis for overall and disease-free survival according to identified risk factors

**Fig. 2:**
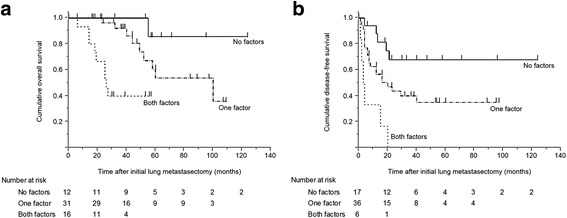
Survival analysis for (**a**) overall survival (OS) and (**b**) disease-free survival (DFS) based on the presence of identified risk factors. **a** Patients with both of the poor prognostic factors for OS (serum carcinoembryonic antigen ≥ 5.0 ng/mL before initial lung metastasectomy and disease-free interval < 24 months after colorectal cancer resection) experienced a significantly shorter OS than those with only 1 risk factor (*P* = 0.039) or no risk factors (*P* < 0.001), although the difference between those with 1 risk factor and no risk factors did not reach statistical significance (*P* = 0.066). **b** Patients who have both risk factors for recurrence (colorectal cancer with N2 stage disease and disease-free interval < 24 months after colorectal cancer resection) had a shorter DFS than those with 1 risk factor (*P* = 0.004) or no risk factors (*P* < 0.001). Patients with 1 risk factor also showed a worse DFS than those with no risk factors (*P* = 0.036)

Considering the results of the multivariate analysis, patients were categorized into 3 subgroups based on whether the patients had a serum CEA ≥ 5.0 ng/mL before lung metastasectomy and a DFI < 24 months after CRC resection, which are 2 unfavorable factors for OS (Fig. 2a). Patients who had both these factors had a significantly worse OS than those with only 1 (*P* = 0.039) or neither of these factors (*P* < 0.001); patients with 1 of the 2 factors tended to have a shorter OS than those with no factors (*P* = 0.066).

Similar analyses were performed regarding DFS, according to the risk factors of a primary CRC with N2 disease and a DFI < 24 months after CRC resection (Fig. 2b). Patients who had both factors experienced a shorter DFS than those with only 1 (*P* = 0.004) or none of the factors (*P* < 0.001); patients with 1 factor also had a worse DFS than those with no factors (*P* = 0.036).

### Distribution of recurrence sites and types of treatments administered in patients with disease recurrence after initial lung metastasectomy

The most frequent site for disease recurrence was the liver (15 cases, 46.9%), followed by the lung (10 cases, 31.3%, including 1 case of stump recurrence). The remaining recurrences occurred as abdominal disseminations (2 cases, 6.3%), in bone (1 case, 3.1%), in mediastinal lymph nodes (1 case, 3.1%), and unknown (3 cases, 9.4%). With regard to treatments after recurrence, surgery alone was performed in 3 cases (9.4%), surgery followed by chemotherapy in 6 cases (18.8%), chemotherapy in 17 cases (53.1%), chemoradiotherapy in 2 cases (6.3%), supportive care in 3 cases (9.4%), and unknown in 1 case (3.1%). More specifically, 5 patients (50.0%) who experienced recurrence in the lung underwent repeated lung metastasectomy.

## Discussion

Numerous studies have previously reported survival after lung metastasectomy for CRC [3, 4, 6, 15–19]. Our study demonstrated a patient 5-years OS of 54.3%, which was similar or slightly better than these previous reports. On the other hand, several prognostic factors after lung metastasectomy for CRC patients were identified, although the results differed slightly among them. Recently, Gonzalez et al. published a review and meta-analysis of this topic, which included 2925 patients and 25 research articles, published from 2001 to 2011 [20]. They concluded that a short DFI after CRC resection, a high serum CEA before lung metastasectomy, multiple lung metastases, and hilar/mediastinal lymph node metastases were poor prognostic factors. Our results are in partial agreement with theirs, but multiple metastases were not found to have a prognostic impact in the present study. This may be due to advances in systemic chemotherapy and the new generation thin-slice CT imaging, although this may be because of a selection bias caused by inclusion of a larger proportion of patients with a solitary metastasis in this study than in previous reports. Further studies are therefore necessary to confirm our findings. Since lung metastasectomy can also be performed safely, it should be conducted proactively even for multiple lung metastases, based on our findings.

The prognostic role of hilar/mediastinal lymph node metastases could not be evaluated in our study because of our criteria for surgical intervention. Several authors have reported a worse prognosis after lung metastasectomy in patients with hilar/mediastinal lymph node metastasis [5, 18, 20, 21]. However, it still remains uncertain whether those patients benefit from lung metastasectomy along with lymph node dissection. As a randomized trial for studying this matter would be difficult to design, the clinicians should predict the possible surgical benefits depending on clinicopathological characteristics in individual patients with hilar/mediastinal lymph node metastasis. The authors basically consider that they should be treated by systemic chemotherapies based on high recurrence rate and poor survival after lung metastasectomy; nevertheless, some patients with limited hilar/mediastinal lymph node metastasis might benefit from lung metastasectomy along with lymph nodes dissection and following adjuvant chemotherapy.

In this study, a 5-years DFS of 40.6% was denoted, which was considerably better than that in past reports [22, 23]. This may be due to clinical advances, such as systemic chemotherapy or thin-slice CT imaging, which allowed more accurate and complete resection. Furthermore, a DFI < 24 months after primary CRC resection and a primary CRC with N2 disease were identified as risk factors for recurrence after initial lung metastasectomy. Few studies have investigated the clinicopathological factors affecting recurrence after lung metastasectomy for CRC. This relative lack of studies may be due to the perceived risk of a variable statistical bias caused by the variety of ways in which cancer cells can spread from a primary CRC. Malignant cells may spread to the lung hematogenously, directly via the inferior vena cava, through the liver via the portal vein, and via the lymphatic pathways. Peritoneal disseminations may also occur, with intra-abdominal dispersion. However, in clinical settings, it is necessarily required to estimate risk of recurrence when performing surgery in which a potential cure is anticipated. Two recent publications have reported factors predicting recurrence, and similar to our study, found that a short DFI after CRC resection was an independent risk factor for recurrence [22, 23]. This suggests that the DFI after a CRC resection strongly predicts the risk of recurrence after an initial lung metastasectomy. Based on these analyses and on our results, we should be aware that patients with a short DFI after CRC resection and a primary CRC with N2 stage disease might be unlikely to attain an extended DFS after lung metastasectomy.

The new generation of chemotherapeutic agents has improved survival of CRC patients considerably. However, most previous studies had not found any survival advantage for the use of adjuvant chemotherapy in patients who have undergone lung metastasectomy, although Park et al. reported an improved DFS in low-risk patients [19]. In fact, our study also did not find a survival benefit of adjuvant chemotherapy for either OS or DFS. This may be because of our patient selection criteria and the various therapeutic protocols that were used for chemotherapy. We believe that survival advantage should be confirmed when adjuvant chemotherapies are administered for more suitable patients. Our results suggest that the administration of adjuvant chemotherapy is best used in patients with these poor prognostic factors.

Parenchymal sparing as the important objective in lung metastasectomy has acquired the consensus, however, the relative high rate of lobectomy was observed in the present study. This is because of some cases with pulmonary central lesion, preoperative diagnosis as primary lung cancer, and multiple lung metastases occupying an entire lobe of the lung. As past studies extensively determined, our study also did not demonstrate significant difference in survival between patients who received wedge resection and anatomical resection. Therefore, it is certain that wedge resection is the top priority as a surgical procedure for lung metastasectomy.

Recent developments of high-resolution CT imaging have enabled the identification of metastatic lesions that would otherwise be too small for gross detection. These advances have allowed a more accurate and complete resection, which has likely contributed to a better prognosis. On the other hand, thoracic surgeons are often exposed to challenging situations in which metastatic lesions seen with imaging studies could not be identified grossly during the operation. Therefore, the minimum size of a metastasis that is indicated for metastasectomy is unclear and often debated. Our results demonstrated that the 5-years OS in patients with a solitary metastasis < 15 mm in diameter had a comparable OS to those patients with lesions ≥ 15 mm (<15 mm, 58.9%; ≥ 15 mm, 51.2%; *P* = 0.656). Although speculative, surgical resection of extremely small, solitary metastatic lesions might be delayed until the lesion size is 15 mm in diameter, to assure a complete and safe resection with an adequate surgical margin.

The limitation of our study is that it included a relatively smaller number of patients than that reported previously. To substantiate the essential prognostic values of each factor, further investigations with a larger number of patients are needed. However, our study focused only on the patients who received treatments after 2004, when the use of modern chemotherapeutic agents began to be administered, which is a valuable finding.

## Conclusion

In summary, our study analyzed patients who underwent initial lung metastasectomy for CRC in modern clinical settings, showing similar or slightly better OS and substantially favorable DFS, as compared with past studies. It appeared that poor prognostic factors for OS did not differ, while the number of lung metastasis was not identified as a poor prognostic factor in the present study; this should be validated in further studies. In addition, initial lung metastasectomy is not expected to be a curable treatment for patients with short DFS after CRC resection and CRCs with N2 stage disease.

## Abbreviations

18^F^-FDG PET: 18^F^-fluorodeoxyglucose positron emission tomography
CEA: Carcinoembryonic antigen
CRC: Colorectal cancer
CT: Computed tomography
DFI: Disease-free interval
DFS: Disease-free survival
MST: Median survival time
OS: Overall survival
